# Supplementary material for: Comparison of soil organic carbon and total nitrogen stocks between farmland treated with three and six years level soil bund and adjacent farmland without conservation measure: In the case of southwestern Ethiopia
Source: PLoS One. 2021 May 27;16(5):e0252123. doi: 10.1371/journal.pone.0252123 (PMC8158939; doi:10.1371/journal.pone.0252123)
Supplement: Attachment — Submitted filename: Response to Reviewers.docx [file pone.0252123.s005.docx]

**Dear Editor-in Chief**

We are very thankful to the anonymous reviewers and Calogero Schillaci for their valuable and constructive comments to amend our manuscript entitled “Comparison of soil organic carbon and total nitrogen stocks between farmland treated with level soil bund and adjacent farmland without conservation measure: in the case of southwestern Ethiopia, ms number “PONE-D-20-35754R1”. We have attempted to address all comments in this revised manuscript and believe the manuscript is now greatly amended. We highlighted **yellow colour** on the **“revised Manuscript with Track Change”** for highlights additional inputs and changes made to the original version. The page where specific responses given for comments are highlighted **red colour** in “**responses to reviewers**” page. Below are our responses to the specific comments raised by the reviewers.

**Queries related to Journal requirement**

Please review your reference list to ensure that it is complete and correct. If you have cited papers that have been retracted, please include the rationale for doing so in the manuscript text, or remove these references and replace them with relevant current references. Any changes to the reference list should be mentioned in the rebuttal letter that accompanies your revised manuscript. If you need to cite a retracted article, indicate the article’s retracted status in the References list and also include a citation and full reference for the retraction notice.

We ensured that all relevant cited papers are listed in the reference list

**Reviewer #1:**

**Abstract Section**

1. Line24-25: the authors said ‘’With respect to the age of LSB, farmland treated with LSB-6 years accumulated more 2.83% SOC stock than control. If they are comparing the overall mean of LSB-6 year to control, normally the corrected value will be 5.83% not 2.83%. Please check it

We excuse for the error and now we changed the value 0f 2.83% to 5.83% (page 1, line 24-25)

**Results and discussion section**

2. Line 203-205 : the authors said that ‘’ The overall mean value of LSB-6 years has exhibited higher mean value of SOC at the lower bund zone (97.81±10.49 Mg ha-1) as compared to LSB-3 years (94.37±7.65 Mg ha-1) (Table 1).’’

Or LSB-6 year exhibited more SOC stock at the lower and middle bund zone

We accepted the comment, and now clarified the sentence in the revised manuscript (page 8, line 197-198)

3. Line 208-211: the authors said “TN socks did not show significant difference (p>0.05) between all inter bund zones. However, under all bund zones, the TN stock showed higher overall mean value under LSB-6 years (9.44±1.57 Mg ha-1) than LSB-3 years (9.28±1.60 Mg ha-1) (Table 1). Similar results were reported by [26] in Zikre watershed, Adaa Berga district. But for LSB-6 year, the TN stock is 9.37±1.10 Mg ha-1 and for the LSB-3 year is 9.44±1.57 Mg ha-1. In this case TN stock showed higher mean under LSB-3 year as compared to LSB-6 year.

Again we are apologized for the error during typing. We have now considerably corrected the mistakes as commented (Page 8-9, line 201-207)

**Reviewer #2:**

1. Lines 139-140 “inter-bund zone …” use the same terminology that you use in the Results section: (upper, middle and lower) instead of (loss, middle and deposition)

Thank you for your comment, now, we rewrote as commented in the revised manuscript (Page 5, Line 133-134)

2. In line 144 “and the same size undisturbed soil samples were collected for bulk density determination” do not repeat 141-142 lines “Similar sample sizes of undisturbed soil were also collected for bulk density determination using core sampler”, just use “bulk density determination”

Now, we rewrote as commented in the revised manuscript (Page 4, Line 104-109)

3. Line 154: bulk density (g/cm3) (use superscript)

We accepted the comments and now, we have used superscript (page 5, line 118)

4. In table 1 do not repeat “SOC stock of/ TN stock of”. Write it only once at the top line of the table and indicate below: “control, LSB-3 years, LSB-6 years”

Thanks for your comment, we rewrote the table heading as commented in the revised manuscript (Table 1)

5. Lines 259-260: “this contribute in mitigating climate change…” According to obtained results it’s better to conclude the deposition of carbon and nitrogen than their sequestering (from the atmosphere). So, I suggest to write “by preventing erosion-induced greenhouse gases emission into the atmosphere” instead of “by sequestering corresponding greenhouse gases”

Thank you for your suggestion, now, we concluded our findings as suggested in the revised manuscript (Page 9, Line 225-226)

**Reviewer #3**

1. The manuscript is proposed as a research article, due to the length (3200 words) the similarity with the previous study (shared experimental settings) of the same work of the authors about SOC %, and the limited novelty, I think it can be proposed as a short communication, a normal research article is made by approximately 6000 works, although I found normal manuscript of 8000 words.

Thank you for your suggestion, even if there are no restrictions on word count for PLOS One, many publishers are guided/recommended the authors as full length Research article and Review article, should not exceed 6000 words. Normally articles will be up to 5000 words including end notes and references. According to those publishers, technical note, Short communication, Comments on a published paper, Corrigendum and Erratum should not exceed 2000 words. (eg. <https://static.springer.com/sgw/documents/1468128/application/pdf/Instructions+for+Authors.pdf>

2. Line24-25: Please check it ‘’With respect to the age of LSB, farmland treated with LSB-6 years accumulated more 2.83% SOC stock than control.” It should be around 5.83%?

We excuse for an error and now we correct the value 0f 2.83% into 5.83% (page 1, line 24-25)

3. Terminology must be consistent throughout the manuscript (e.g. lines 139-140 inter-bund zone), please check the consistency.

Thank you for your comment, now, we rewrote as commented in the revised manuscript (Page 5, Line 133-134)

4. I would rephrase the sentence “by sequestering corresponding greenhouse gases” in som I would rephrase the sentence “by sequestering corresponding greenhouse gases” in something more neutral. Please define better the conclusion of the experiment. Do not oversell the findings.

Thank you for your suggestion, now, we concluded our findings as suggested by anonymous reviewer in the revised manuscript (Page 9, Line 229-230)
